# Non-Surgical Approaches to the Management of the Intrathoracic Goiter—A Systematic Review

**DOI:** 10.3390/jpm14111079

**Published:** 2024-10-29

**Authors:** Cesare Miani, Luca Giovanni Locatello, Nicole Caiazza, Anna Maria Bergamin-Bracale, Stefania Rigo, Maria Gabriella Rugiu, Andrea Zuin, Ricard Simo

**Affiliations:** 1 Department of Otorhinolaryngology, Academic Hospital “Santa Maria Della Misericordia”, Azienda Sanitaria Universitaria Friuli Centrale, Piazzale Santa Maria Della Misericordia 15, 33100 Udine, Italy; cesare.miani@uniud.it (C.M.);; 2 Department of Medicine (DAME), University of Udine, Via Colugna 50, 33100 Udine, Italy; 3 Department of Otorhinolaryngology, Sant’Antonio Abate Hospital, Azienda Sanitaria Universitaria Friuli Centrale, 33028 Tolmezzo, Italy; 4 Department of Thoracic Surgery, Academic Hospital “Santa Maria Della Misericordia”, Azienda Sanitaria Universitaria Friuli Centrale, Piazzale Santa Maria Della Misericordia 15, 33100 Udine, Italy; 5 Department of Otorhinolaryngology Head and Neck Surgery, Guy’s and St Thomas’ Hospital NHS Foundation Trust, King’s College London, London SE1 7EH, UK

**Keywords:** goiter, embolization, radiofrequency, head and neck, thyroid surgery

## Abstract

Background: Intrathoracic goiters (ITGs) are usually managed by surgical excision, However, over recent years, non-surgical strategies are emerging as an alternatives for treating this condition. Methods: A systematic review of research published since 2017 in the PubMed database was conducted and a total of 39 articles were retrieved, along with methodological issues and future directions in the research on ITGs. Results: Several non-surgical treatments exist, including radio-iodine ablation (RIA) and mini-invasive approaches, such as transcervical microwave ablation (TcMA), transcervical radiofrequency ablation (TcRfA), or selective embolization of the thyroid arteries (SETA). Despite encouraging reports, their current use remains limited. Conclusions: Treatment of ITGs requires a multidisciplinary thyroid team, and when non-surgical options are chosen, patients need to be carefully selected, and their outcomes must be prudently considered and discussed with the patient.

## 1. Introduction

Intrathoracic goiters (ITGs) have been traditionally addressed with total surgical excision, primarily by transcervical, transthoracic, or combined approaches [1,2]. Their surgical efficacy has been extensively reported in the literature [3,4,5]. In the last few years, new findings on the biological and clinical aspects of this condition have also been published, and there is growing evidence that non-surgical strategies for treating ITGs could be considered. These include, amongst others, radio-iodine ablation and new mini-invasive techniques, such as image-guided ablation or transarterial embolization [5,6]. 

The aim of this paper is to report and discuss the evidence published in the last six years on the clinical aspects and medical (non-surgical) management of ITGs via a systematic review method.

## 2. Materials and Methods

**Figure 1:**
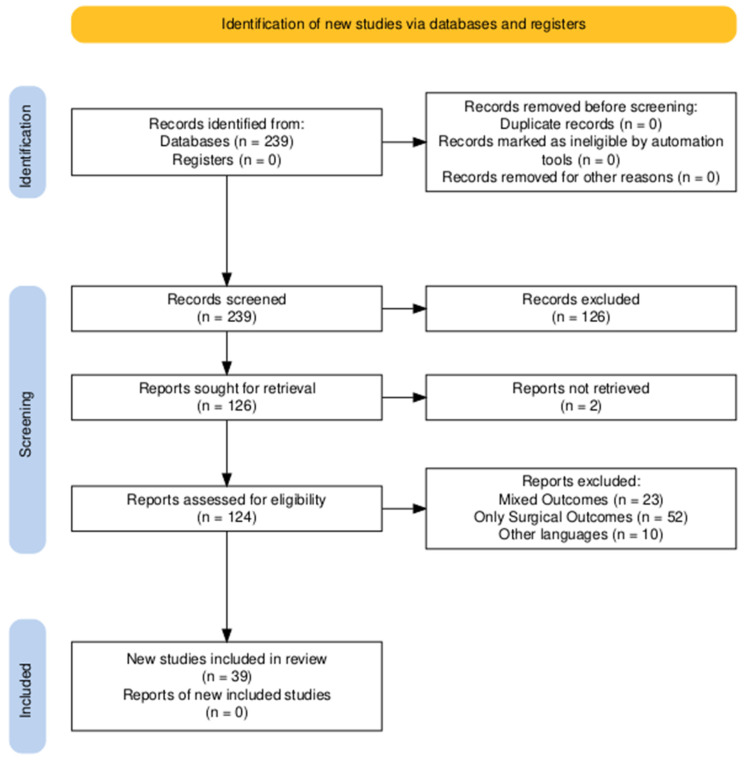
A modified PRISMA flowchart for selection and inclusion of the relevant studies in the present review.

The PRISMA statement was followed in the preparation of the present paper, and a modified PRISMA flowchart is given in Figure 1 [7]. No institutional review board approval was necessary for the present work since it does not involve patients. This was an unregistered systematic review. The PICOS methodology was used, with the main question being “What are the current non-surgical treatment options for managing intrathoracic goiter?”. In detail, P (patients)—patients diagnosed with ITGs; I (intervention)—non-surgical treatments; C (comparator)—none; O (outcomes)—resolution of symptoms and rates of posttreatment complications; S (study design)—reports, retrospective or prospective cohort studies, and randomized clinical trials.

The PubMed database was used in order to perform the review of the literature from 1 January 2017 to 1 January 2024. The following search string was used: “(intrathoracic OR retrosternal OR cervico-mediastinal OR substernal) AND (goiter OR goitre)”. All the pertinent articles were included after careful reading of the titles and abstracts. The full texts of the included articles were then retrieved by the first co-author (LGL) and quantitative and qualitative data were synthesized accordingly. The following papers were excluded: papers reporting on the outcomes of ITGs mixed with other thyroid disorders (reason 1); articles reporting on the surgical outcomes of ITGs (reason 2); papers written in languages other than English, French, or Italian (reason 3).

The search strategy retrieved a total of 239 articles and, after applying the selection criteria, a total of 39 articles were ultimately analyzed. Additionally, a further article was retrieved and included in the discussion after checking through the reference lists of the relevant studies. Quantitative and qualitative data regarding surgical outcomes were summarized and systematically reported in tables.

## 3. Results

### 3.1. Pathophysiology of ITGs

Regarding the biology and pathogenesis of ITG, no recent study has explored the question of whether there are different molecular mutations in ITGs compared to non-intrathoracic goiters. Anatomical and functional factors (no limitations encountered by the downwards-expanding gland, traction forces associated with swallowing, a negative intrathoracic pressure during inspiration) and the force of gravity itself have been called into question [4]. A 1939 study by Crile proposed that patients with a short neck and hypertrophic cervical muscles might theoretically favor the intrathoracic expansion of the goiter [8], but no formal anthropometric study exists to corroborate this assumption, even in the most recent papers. Other extremely rare presentations include presternal goiters [9] or intrapericardial (the 7th case was published in 2019) goiters [10], and embryological remnants attached to the aortic arch might explain these pictures. In the end, and besides the simple time during which a goiter has been allowed to grow undisturbed, risk factors for the development of ITGs remain unexplored. In a biochemical investigation on the oxidative state in normal and tumoral thyroidal tissues, a research group found that patients with ITGs have lower malondialdehyde levels compared to non-retrosternal goiters. Since this compound is a pro-oxidative marker, the authors conclude that ITGs show a “better antioxidative potential”: this is attributed to the scavenging ability of the largely present inorganic colloid, but the clinical meaning of this finding remains puzzling [11]. In addition, there are very rare cases where engorgement of the gland is not due to colloidal production. An example is given by amyloid goiters, and treating the underlying systemic condition is necessary after the diagnosis on the surgical specimen [12]. Another rarer lesion is diffuse thyroid lipomatosis, which can be suspected by the signal on CT scans and that is characterized by diffuse fatty infiltration in thyroid stroma on histopathology [13].

### 3.2. Clinical Presentation

A chronic and slowly progressive airway compression is the usual presentation of ITG, and this can last many years, especially in areas where iodine deficiency is endemic [14]. A choking sensation exacerbated by a supine position, globus, wheezing, and exercise-induced dyspnea (both mistaken for asthma) or dysphagia are the symptoms most commonly reported by patients; these are asymptomatic at presentation in over one-third of cases [15]. Work by Rodrigues et al. from Brazil has investigated the prevalence of signs of laryngopharyngeal reflux in ITG patients [16]. Using findings on preoperative laryngoscopy, they found reflux to be significantly more frequent in ITG patients compared to simple goiter patients (*p* = 0.036) [16]. This work is retrospective, and the diagnosis of reflux laryngitis was not conducted by a validated endoscopic score. Nonetheless, the association has two plausible pathophysiological mechanisms: first, the authors of the study believe that esophageal peristalsis could be impaired by possible compression of the vagus nerve against the first rib. Secondly, a voluminous ITG would cause an esophageal narrowing that would favor bolus accumulation in the upper part of the esophagus [16]. These remain unproven theories, but they indeed deserve more attention in future studies. 

The mechanisms of goiter-associated dyspnea are believed to be direct extrinsic tracheal compression, dysfunction of the recurrent laryngeal nerve, and lung atelectasis [17,18]. Another potential, yet extremely rare, cause of dyspnea is given by tracheobronchopathia osteochondroplastica. The latter is segmentary tracheal stenosis caused by inflammatory osteocartilaginous nodules, and that is diagnosed by chest computed tomography along with bronchoscopy: since some authors report an association with concomitant ITGs, this condition should not be overlooked in the radiological workup [19].

Instead, in less than 5% of cases, the clinical presentation of ITG may be acute, and spontaneous hemorrhagic enlargement of ITG may occur during pregnancy [20,21] or for unclear reasons [22,23]. Acute asphyxia and cardiac arrest have been described [24,25], and some authors have hypothesized a direct phrenic nerve compression by the RG as the main cause of these dramatic scenarios [26].

Much rarer presentation may be constituted by thyrotoxicosis or symptoms of superior vena cava syndrome, that is facial plethora and congestion, upper neck cyanosis, or a positive Pemberton’s sign with facial flushing and engorgement of superficial jugular veins upon raising the arms [15]. While it had been previously reported that this syndrome was more often associated with malignancy, this remains unproven [4]. Isolated dysphagia is also possible in case of goiter growing only in the retro-esophageal plane [27,28], while the only presenting symptom may also be represented by oropharyngeal bleeding because of pharyngolaryngeal “downhill” (i.e., without venous portal hypertension) varices [29]. Furthermore, lower and upper extremities edema [30], pericardial effusion, chylothorax, or hemoptysis because of tracheal varices have been also described [4]. These symptoms are common to all kinds of mediastinal masses, where thymomas and lymphoma are much more common than purely retrosternal (type III) goiter. Differential diagnosis is imperative and FNAC showed an excellent diagnostic performance when feasible (90% of cases in a recent series [31]).

### 3.3. Non-Surgical Management of ITG: Medical Therapy

When surgical excision is not indicated or it is not feasible due to patient’s frailty, or comorbidities, a wait-and-see approach or medical treatment of ITG may be proposed. This is certainly true for those patients in which ITG is an incidental finding and it does not cause any clinical symptoms [4,15].

In the most recent literature, no new studies regarding levothyroxine supplementation or radioactive iodine (RAI) treatment have been published. For both options, many drawbacks are present (missed malignancy, risk of thyrotoxicosis, or increased airway obstruction) and only with the advantage of a slow and modest size reduction at best [32]. There is old evidence that shrinkage is not proportional to the substernal size and the median intrathoracic volume is reduced by 29.2% (range, −6.1–59.4%, mean: 26.1% +/− 6.0%) [33]. The latest 2023 guidelines from the European Association of Nuclear Medicine state that RAI can be considered for non-toxic ITGs “when surgery cannot be performed or is refused by the patient” [34]. However, among the relative contraindications, we found the criterion of patients presenting with “very large goiters (≥80 g or >100 mL)” [34]. There is a recent report of a successful treatment by RAI of a spontaneous chylothorax associated with a hyperfunctioning ITG, because the patient had refused surgery [35]. According to the authors from South Korea, in several weeks after the administration of the radioactive agent (and the application of a pleural drainage), there was an improvement in compressive symptoms, for thyrotoxicosis, and with the resolution of the pleural opacity [35].

### 3.4. Transcervical Ablative Techniques

In the last decade, there has been an ever-growing interest in interventional ablative techniques for thyroid nodules and cysts [36]. As recently reviewed by Kuo et al., several scientific societies have endorsed their use as an alternative to partial/total thyroidectomy for benign and selected malignant lesions (including malignant lymph nodes) [37]. In particular, percutaneous ethanol ablation is a first-line option for recurring cystic nodules, and several thermal ablation techniques (such as puncture-less high-intensity focused ultrasound, laser ablation, transcervical microwave ablation (TcMA), or transcervical radiofrequency ablation (TcRfA)) have shown efficacy in several studies [36,37]. All these interventional ablation techniques may be safely performed in an outpatient setting, are well tolerated, and the risk of needing thyroid hormone supplementation is almost negligible [37]. Periprocedural complications are also very limited (pain, neck discomfort, hematomas), even though rarely vocal cord paralysis or even thyro-cutaneous fistula formations ultimately requiring surgery have been described [38]. As these approaches are becoming a popular alternative treatment to surgical resection, the size of the nodule remains a key criterion. For benign nodules, TcRfA is best used in nodules up to 30 mm, while for documented malignancies, guidelines suggest limiting it to nodules under 20 mm (i.e., cT1b differentiated cancer) [36]. For large nodules (approximately volume ≥20 mL or diameter ≥50 mm), these techniques are usually discouraged, although repeated ablations are proposed by some authors with good results [39]. Consequently, ITGs were not even considered in this regard until a few years ago when minimally invasive techniques such as TcMA, TcRfA, or selective embolization of the thyroid arteries (SETA) started to be explored further [40,41,42,43]. An overview of the published studies is presented in Table 1.

#### 3.4.1. Transcutaneous Microwave Ablation (TcMA)

Ultrasound-guided TcMA of ITG combined with ethanol injection was reported as an effective and safe treatment for solitary nodular ITG, especially for patients who are ineligible or unwilling to receive surgical treatment [40,41]. A first preliminary experience was presented by Cui et al. in 2018 in a cohort of 10 patients. A mean volume reduction ratio (VRR) of around 67% was reported one month after the procedure. No local complications (pain, hematoma, etc.) or postoperative functional adverse events (thyrotoxicosis crisis, changes in TSH, etc.) were reported [40]. A subsequent prospective study from the same hospital involved 72 patients with uninodular ITGs, with the aim of treating only that part by TcMA. The cohort had a mean age of 47.8 years, and the mean follow-up time was 23.9 months. Notably, TcMA yielded a mean VRR of 83.12 ± 12.74% (range: 52.01–100%) after one year. A total of 57 patients (79.2%) showed complete regression of the intrathoracic extension (as measured by CT scan), while 8 patients (11.1%) needed a second procedure because of “regrowth of unsatisfactory reduction”. Two patients reported temporary neck pain/discomfort, while another one developed postprocedural dysphonia, which resolved spontaneously 1 month following the event, for an overall complication rate of 4.2% [41]. It should be noted that, in both studies, a significant reduction in terms of ITG-associated signs and symptoms (i.e., neck circumference and a VAS scale measuring pain, shortness of breath, positional dyspnea, dysphagia, and dysphonia) was obtained, even though the procedure focused only on the largest retrosternal nodule [40,41].

#### 3.4.2. Transcutaneous Radiofrequency Ablation (TcRfA)

TcRfA is another recently described method. Chiang et al. recently reported a VRR of 75.5% (*p* < 0.001) in a series of 16 ITGs. In this series, only four (25%) patients showed complete regression of the intrathoracic portion of the goiter, with one patient developing temporary hoarseness and another one developing subcutaneous/focal mediastinal hematoma, which was managed conservatively (complication rate 12.5%). Nonetheless, all the patients reported significantly lower cosmetic and symptom scores on the VAS scale at 6 months [42]. From a technical point of view, all the procedures were performed under local anesthesia; hydrodissection in the danger triangles around the course of recurrent laryngeal nerves was always performed at the beginning. The most striking result of this work is the response of the intrathoracic part of the goiter, as assessed by a CT scan. Before TcRfA, the mean centimeters and proportion (%) of the retrosternal part with respect to the total goiter volume were on average 1.55 cm (18.1%), with a range from 0.38 to 2.85 cm (5.44 to 40.83%). After the procedure, this interval was 0–2.55 cm (0–34.69%) and with a mean substernal part of 0.97 cm (12.97%), which is a mean significant proportional reduction of 5%. These figures are encouraging but the authors also performed a subanalysis of the ITG cases stratified by the CT cross-sectional imaging (CSI) classification system [44]. Briefly, this divides the ITGs according to the extension into the cranio-caudal plane of the lower margin (grades 1–3), and of the anteroposterior relationships of the ITG (types A–C) [44]. If we look at the results in terms of staging, not unexpectedly, patients with the smallest and most accessible ITGs (classified as 1 A) had the best treatment responses. Instead, for the upper categories, this benefit was negligible at best, thus confirming some technical limits of the procedure and stressing again the importance of using a staging system [42]. 

#### 3.4.3. Selective Embolization of the Thyroid Arteries (SETA)

Regarding the SETA procedure, a limited Italian series of 10 patients reported complete success in reducing the thyroid function and ITG dimensions but no formal endpoints were used, and follow-up time was not reported. In addition, all patients developed transient thyrotoxicosis requiring corticosteroids, antibiotics, and methimazole, while one patient (10%) had right vocal cord fixation lasting around 6 months [43]. Yilmaz and coworkers instead presented their experience with SETA in 56 goiters; among them, 47 showed “some degree of intrathoracic extension, which was the main reason for preferring embolization over other treatments” [6]. This inclusion criteria seems liberal given the potential risk of inadvertent reflux embolization to the brain, due to the proximity of the carotid and vertebral arteries. Another point to be considered is that SETA does not have the advantage of local anesthesia as other procedures do. On the contrary, because the entire gland is addressed by the embolization, there are no limits in the case of ITG. The procedure was conducted successfully by authors in 145 out of 146 thyroid arteries and complications occurred in 27 patients (48%), but if we eliminate a case of intraoperative blurry vision, 1 case of groin hematoma, and 23 cases of thyrotoxicosis, only 3 (5%) patients developed temporary hoarseness. Notably, after 6 months, the mean thyroid volume was reduced from 147.0 mL to 62.6 mL, while the mean retrosternal extension was reduced from 31.7 mm to 15.9 mm (*p* < 0.001). Quality of life was measured by the popular ThyPRO tool and the mean scores improved from 155.4 to 70.4 (*p* < 0.001) [6]. These authors also reported that one patient died of myocardial infarction two weeks after SETA, and a possible causative effect cannot be excluded. Despite the absence of a comparative study, SETA is reported to be applicable irrespective of the goiter dimensions, but this does not fit the preprocedural reported mean dimensions of the nodules (80.2 ± 46.7 mL for SETA [6], versus 76.10 ± 50.56 mL for TcMA [41]).

## 4. Discussion

All these non-surgical approaches present many advantages (no reported risk of iatrogenic hypothyroidism or hypoparathyroidism; they are a day or one-day procedures for ablation and SETA, respectively; they can be performed even in very fragile patients, etc.). However, they retain the fundamental limit of not permitting any histological evaluation of the ITG, even though at least one [6] or two [41] consecutive non-malignant (up to Bethesda category 3) fine-needle core biopsies were always required as an inclusion criterion. Another limitation of these techniques is that they often ablate only the intrathoracic nodule(s) rather than the entire gland. With a longer follow-up, we do not know the kinetics of regrowth of the remaining gland. Given the limited observation of these cohorts and the current life expectancy, it remains unclear whether these procedures can be repeated. In case a subsequent surgical rescue is decided, it also remains to be seen whether patients are at a higher risk of complications given the predictable fibrosis induced by the ablation. Most importantly, the lack of a head-to-head comparison between standard surgical resection and these techniques remains the key methodological drawback. Finally, most of the studies suggest these techniques to be an alternative to thyroidectomy whereas, in the future, it would be much more interesting to consider them as a method to avoid extracervical surgical approaches. We should also emphasize that, in some cases (for instance, asymptomatic nodules less than 3 cm in old patients), the need for any treatment should be thoroughly discussed with the treating physician.

Our work has yielded an updated overview of the current non-surgical strategies for managing ITGs, and we hope that it may represent the basis for developing new research ideas, protocols, and guidelines. Limitations of our paper include a potential search and eligible study identification bias because only PubMed was used. However, this must be confronted with the fact that many databases do not adequately perform as a main source for systematic search [45].

## 5. Conclusions

The management of ITGs continues to be based on surgical resection, yet several alternatives have emerged in the last few years. A formal comparison of the outcomes of such treatment strategies starts from a uniform definition of what is actually an ITG and of the use of validated classification (staging) systems. An understanding of ITG biology and the natural course of this condition remains to be determined. Future insights into the pathophysiology of ITGs will also help us to understand the growth mechanisms and possibly enable the discovery of some pharmacological targets. The above-described non-surgical treatments are nonetheless encouraging and they could represent a real alternative where the surgical options are not possible. Based on our literature search, current data are too scarce to identify the “best” minimally invasive procedure, and future studies need longer follow up time. With continuing developments in non-surgical treatments further research in this field should be encouraged.

## Figures and Tables

**Table 1:** An overview of the most recent publications reporting non-surgical treatments for intrathoracic goiters. FNAC, fine needle aspiration cytology; NA, not available; RLNP, recurrent laryngeal nerve palsy; US, ultrasound; VRR, volume reduction rate.

Reference and Country	Number of Procedures	Mean Follow-Up Time	Definition of ITG	Inclusion and Exclusion Criteria	Mean Volume of the ITG, Technique Used for Measurement	Technique Used	Type of Anesthesia	Mean Duration of the Procedure	Overall Success Rate	Overall Complication Rate
Chiang et al., 2021, Taiwan [42]	16	12.5 months	“non-visualized inferior margin of the thyroid nodule by US”	Included: (1) age ≧ 18 (2) benign ITG by FNA (Bethesda ≦ 2). Excluded: (1) hypothyroidism or subclinical hypothyroidism, (2) patient with a pacemaker, and (3) pregnant people	Mean goiter volume as measured by US was 106.62 mL (range 29.2–252; SD 61.82)	Transcutaneous radiofrequency ablation	Local anesthesia (100%)	Mean procedural time was 74.13 min (range 49–103; SD 17.12)	25%	12.5%
Cui et al., 2019, China [40]	10	4 months	“an enlarged thyroid gland descending below the thoracic inlet, with the diagnosis based on radiologic evidence”	Included: (1) symptomatic compression (2) the lower pole of the SSG extending no greater than 3 cm below the plane of the thoracic inlet; (3) refusal of or ineligibility for surgery; (4) benign FNAC. Excluded: (1) malignant signs on US imaging	Mean volume of the nodules was 52.9 +/+ 27.9 mL (range, 23.7–122.6 mL), by US	Transcutaneous microwave ablation	Local anesthesia (100%)	28.2 min ± 9.4 min (range, 16.5–41.1 min)	Nodule volume reduced at the 3-month follow-up (17.5+/− 9.5 mL; *p* < 0.05). Three-month VRR of the index nodule was 66.7% +/− 7.1%	0
Li et al., 2023, China [41]	72	23.89 months ± 7.66 months (range 15–39 months)	“goiter descending below the plane of the thoracic inlet”	Included: (1) symptomatic solitary nodular RSG diagnosed with CT images; (2) benign pathology determined by at least two FNAC; (c) euthyroidism; and (d) ineligible or unwilling to receive surgery. Excluded: (a) history of neck irradiation; (b) age < 18; (c) pregnancy; and (d) loss of follow-up	Mean nodule volume was 71.25 mL ± 61.61 mL	Transcutaneous microwave ablation	Local anesthesia in 100%	22.46 min ± 3.72 min (range 15.25–32.00 min)	Volume decreased significantly to 7.47 mL ± 9.19 mL; a mean VRR of 90.99% ± 7.52%	4.2% (3/72). Two local pain; one transient hoarseness
Tartaglia al., 2019, Italy [43]	10	NA	“voluminous cervico-mediastinal goiter located deep in the mediastinum, below the horizontal plane passing through the aortic arch”	Included: (1) ITGs and significant comorbidities contraindicating surgery. Excluded: not stated	NA	Selective embolization of the thyroid arteries	General 100%	NA	70% (three required subsequent thyroidectomy)	20% 1 case of permanent RLNP + 1 case of transient RLNP
Yilmaz et al., 2021, Turkey [6]	47	NA	NA	Included: (1) Single nodules not eligible for percutaneous laser or radiofrequency ablation (>5 cm in size, intrathoracic extension) and multiple nodules with compressive or cosmetic symptoms; (2) a Bethesda category 2–3 on FNAC. Excluded: (1) renal insufficiency; (2) inability to tolerate angiography	31.7 ± 19.8 (12–90)	Selective embolization of the thyroid arteries	General 100%	NA	Mean reduction in the intrathoracic part from 31.7 ± 19.8 (12–90) mm to 15.9 ± 10.3 (5–50) mm at six months	22/47 minor events, 2/47 major (hematoma and thyroid storm), 1 deceased from myocardial infarction after two weeks

## Data Availability

No new data have been generated.
